# Relative Age Effects in Athletic Sprinting and Corrective Adjustments as a Solution for Their Removal

**DOI:** 10.1371/journal.pone.0122988

**Published:** 2015-04-06

**Authors:** Michael Romann, Stephen Cobley

**Affiliations:** 1 Department of Elite Sport, Swiss Federal Institute of Sport, Magglingen, Switzerland; 2 Exercise & Sport Science, Faculty of Health Sciences, The University of Sydney, Sydney, Australia; University of Buenos Aires, Faculty of Medicine. Cardiovascular Pathophysiology Institute., ARGENTINA

## Abstract

Relative Age Effects (RAEs) refer to the selection and performance differentials between children and youth who are categorized in annual-age groups. In the context of Swiss 60m athletic sprinting, 7761 male athletes aged 8 – 15 years were analysed, with this study examining whether: (i) RAE prevalence changed across annual age groups and according to performance level (i.e., all athletes, Top 50%, 25% & 10%); (ii) whether the relationship between relative age and performance could be quantified, and corrective adjustments applied to test if RAEs could be removed. Part one identified that when all athletes were included, typical RAEs were evident, with smaller comparative effect sizes, and progressively reduced with older age groups. However, RAE effect sizes increased linearly according to performance level (i.e., all athletes – Top 10%) regardless of age group. In part two, all athletes born in each quartile, and within each annual age group, were entered into linear regression analyses. Results identified that an almost one year relative age difference resulted in mean expected performance differences of 10.1% at age 8, 8.4% at 9, 6.8% at 10, 6.4% at 11, 6.0% at 12, 6.3% at 13, 6.7% at 14, and 5.3% at 15. Correction adjustments were then calculated according to day, month, quarter, and year, and used to demonstrate that RAEs can be effectively removed from all performance levels, and from Swiss junior sprinting more broadly. Such procedures could hold significant implications for sport participation as well as for performance assessment, evaluation, and selection during athlete development.

## Introduction

The practice of annual age grouping occurs throughout and across youth sport and education. In sport, administrators typically categorise participants into annual age groups for logical logistical control purposes, and to reduce developmental differences during childhood and adolescence [1] in an attempt to help maintain a more equal and even playing-field. In regards to the latter, an unfortunate problem remains in that there is potential for up to 12 months of chronological age difference—and potentially more in terms of biological age difference—between individuals within an annual age-group cohort. These can lead to outcomes known as Relative Age Effects (RAEs) [2]. RAEs reflect the interaction between an athlete’s birth date and the dates used for chronological age grouping, and whereby being relatively older compared to being relatively younger, generates consistent participation inequalities, selection biases, and attainment advantages in developmental ages and stages of sport [1].

RAEs are most highly prevalent across numerous male team sport contexts, and less consistently evident in female sport contexts [1,3]. For instance, participation ratios between the relatively oldest and youngest quartiles of annual-age groups have varied between 1.5 to as high as 9 to 1. These figures relate to studies in contexts of school, local junior league, representative, and youth international soccer [4,5], baseball [6], handball [7], both codes of rugby [8] and Australian rules football [9]. More recently, studies have also identified that individual, but still physically demanding sports are also affected. These include tennis [10], swimming [11], ski-jumping, cross-country, and alpine skiing [12,13], as well as a variety of other strength, endurance, and technique based events, as identified in a study of participants in the Youth Winter Olympic Games [14]. By contrast, sport contexts with a skill emphasis such as golf [15], and with less dependence on physical characteristics, appear immune to RAEs in participating samples; while the association between RAEs and dropout seems contextual and inconsistent [16,17].

Several inter-related hypotheses have been proposed to explain RAEs, but most prominently supported is the ‘maturation-selection’ hypothesis [1], which states that greater chronological age is equated with an increased likelihood of enhanced anthropometric characteristics from normative growth and development. Greater height and lean body mass are predictive of better physical capacities such as aerobic power, muscular strength, endurance and speed [18], so in turn these characteristics provide physical performance advantages in most sport tasks [19]. Also, during maturation, the relatively older are more likely to enter puberty earlier, and the tempo of maturation may generate further anthropometric and physical variation between individuals until its cessation [20]. Thus in the short-term, the relatively older and earlier maturing are more likely to be considered as better athletes, and be selected by coaches for higher levels of competition. Unfortunately, the relatively younger and later maturing are more likely to be overlooked and excluded [21] in the various participation stages of junior and youth sport, at least until the end of growth and maturation. The hypothesis thus can also account for why RAEs, albeit with smaller effect sizes, lag into adult and professional sport contexts.

With few studies to date examining individual sport contexts or testing underlying mechanistic hypotheses of RAEs; and, fewer still identifying or offering potential feasible solutions to eliminate RAEs (see Cobley et al., [1] for a summary) and their detrimental impact on sport participation and experience, we considered how an investigation of an athletics contexts could provide beneficial insight. Athletics has only partially been considered in RAE literature [3], yet events such as distance running, sprinting, and long jump generally demand advanced physical capabilities like high VO2 max; lower-leg muscle mass, strength and power for performance success, with lesser concern for extraneous or confounding inter-athlete variables like team formations, tactics, positional roles and selection as occurs in team sport contexts [9,22]. So RAEs should be hypothetically prevalent due to the benefits of advanced relative and biological age. Performance here can also be more objectively measured in terms of Centimetres, Grams, and Seconds (i.e., CGS Sports [23]), and quantifiable relationships between relative and chronological age and performance can be estimated. This then permits an assessment of whether the ‘maturation-selection’ hypothesis can consistently explain RAE outcomes across junior and youth athlete development, and whether this could be statistically controlled with a corrective adjustment procedure tested for RAE removal.

In Switzerland, track and field is the most popular individual summer sport [24]. For instance, in the 2013 season, 7761 male children and adolescents aged 8 – 15years participated in an official 60m sprint trial, and so this provided an appropriate context to firstly determine whether RAEs were prevalent within and across junior/youth sprinting, affecting participation and performance, and whether RAEs were amplified at higher performance levels. Then, relationships between relative age, chronological age, and physical performance could be determined; subsequently allowing us to test and apply a corrective adjustment procedure to remove RAEs.

## Methods

### Participants

This study was approved by an independent institutional ethical review board of the Swiss Federal Institute of Sport Magglingen, Switzerland and is in accordance with the principles expressed in the Declaration of Helsinki. Informed consent was not needed as the study analyzed and reported data was available online. However, all data is reported anonymously. Participants were *N* = 7761 male Swiss youth track and field 60m sprint athletes, aged 8–15, who participated in an official local, regional, or national trial event, and whose performance was recorded using electronically timed photo sensors (ALGE Timing OPTIc2, Switzerland). All recorded sprints conformed to standards of the International Association of Athletics Federations. Trials took place either in schools or track and field clubs and were open to all. An official registration or licence process was not required. During the 2013 competitive season, the personal best performance time, birth date, age group, and name of each participating athlete was recorded in the database of the Swiss Athletics Federation [25].

### Part 1—procedures

For part one, participant age, date of birth, and sprint times were examined across the ages of 8 to 15. To determine whether RAEs existed, athletes were categorised according to annual-age year group and relative age quartile. For all track and field events in Switzerland, January 1^st^ acts as the cut-off date for age-grouping; so, with this as a reference, athletes were ascribed to one of four relative age quartile categories (i.e., Q1 = born in January–March; Q2 = April–June; Q3 = July–September; and, Q4 = October–December). Relative age distributions across all athletes and age groups were then calculated and referenced against actual corresponding birth-distributions from the Swiss population using weighted mean scores. The corresponding Swiss population aged 8–15 years was defined as the number of official male residents (*n* = 290, 977) registered with the Swiss Federal Statistical Office [26]. All relative age quartiles were approximately equally distributed (e.g., male: Q1 = 24.7%; Q2 = 25.2%; Q3 = 26.0%; Q4 = 24.1%), and these exact distributions in the broader population were used in data analyses. Within each age group, the sample was then subdivided into the fastest or Top 50%, 25% and 10% of sprint performance respectively to assess whether RAE effect sizes were related to performance level (Table 1).

**Table 1:** RAE as a function performance level and annual age-group category.

Performance Level	Age Group	*n*	Q1 (%)	Q2 (%)	Q3 (%)	Q4 (%)	*χ* ^*2*^	*P*	V	Effect	OR	*P*	95%CI
all	8	578	33.4	27.2	23.9	15.6	37.8	**	0.15	small	2.11	*	(1.64–2.71)
	9	824	32.0	25.4	24.6	18.0	32.4	**	0.11	small	1.75	*	(1.43–2.15)
	10	1267	30.4	26.4	24.9	18.3	36.8	**	0.10	small	1.63	*	(1.38–1.93)
	11	1370	27.7	25.9	26.8	19.6	18.3	**	0.07	small	1.39	*	(1.19–1.64)
	12	1356	27.8	26.8	25.1	20.4	15.7	**	0.06	small	1.34	*	(1.15–1.60)
	13	1238	26.7	25.8	25.8	21.7	5.5		0.04	no	1.21	*	(1.02–1.42)
	14	649	30.4	23.3	25.9	20.5	13.4	**	0.08	small	1.46	*	(1.17–1.82)
	15	479	33.8	23.2	21.5	21.5	22.5	**	0.13	small	1.55	*	(1.21–1.98)
	8–15	7761	29.5	25.8	25.2	19.6	147.0	**	0.08	small	1.48	*	(1.16;1.90)
top 50%	8	288	43.4	26.7	20.5	9.4	71.2	**	0.28	medium	2.16	*	(1.42–3.28)
	9	412	38.8	26.7	22.8	11.7	62.7	**	0.23	medium	1.87	*	(1.35–2.58)
	10	633	33.5	29.4	23.9	13.3	57.1	**	0.17	medium	1.52	*	(1.18–1.96)
	11	685	33.6	27.6	25.0	13.9	54.5	**	0.16	small	1.71	*	(1.34–2.17)
	12	678	34.2	27.3	22.4	16.1	48.5	**	0.15	small	1.56	*	(1.24–1.60)
	13	619	33.9	27.8	19.7	18.6	41.0	**	0.15	small	1.49	*	(1.18–1.87)
	14	324	36.7	25.6	20.4	17.3	29.8	**	0.17	medium	1.43	*	(1.04–1.97)
	15	239	41.0	22.6	17.2	19.2	36.4	**	0.23	medium	1.35		(0.95–1.92)
	8–15	3878	35.7	27.2	22.1	15.0	361.9	**	0.18	medium	1.59	*	(1.44–1.76)
top 25%	8	144	44.4	32.6	14.6	8.3	48.4	**	0.33	large	2.49	*	(1.34–4.61)
	9	206	44.2	27.7	17.0	11.2	53.5	**	0.29	large	2.22	*	(1.40–3.51)
	10	316	38.3	33.2	19.9	8.5	68.6	**	0.27	medium	2.70	*	(1.78–4.10)
	11	342	36.0	30.4	24.0	9.6	52.1	**	0.23	medium	2.63	*	(1.79–3.86)
	12	339	41.9	26.8	20.6	10.6	71.1	**	0.26	medium	2.89	*	(2.00–4.17)
	13	309	39.5	28.8	18.1	13.6	51.2	**	0.23	medium	2.37	*	(1.67–3.37)
	14	162	45.1	25.3	17.9	11.7	42.1	**	0.29	large	2.59	*	(1.56–4.30)
	15	119	42.9	21.8	17.6	17.6	22.0	**	0.25	medium	1.54		(0.93–2.57)
	8–15	1937	40.6	28.9	19.5	11.0	384.3	**	0.26	medium	2.45	*	(2.10–2.86)
top 10%	8	57	49.1	29.8	12.3	8.8	24.15	**	0.38	large	2.61	*	(1.01–6.77)
	9	82	52.4	24.4	13.4	9.8	37.9	**	0.39	large	3.01	*	(1.42–6.41)
	10	126	40.5	34.1	19.0	6.3	35.8	**	0.31	large	3.84	*	(1.82–8.10)
	11	137	41.6	28.5	24.1	5.8	36.0	**	0.30	large	5.03	*	(2.40–10.54)
	12	135	45.2	25.2	18.5	11.1	35.7	**	0.30	large	2.98	*	(1.69–5.24)
	13	123	46.3	24.4	18.7	10.6	35.6	**	0.31	large	3.57	*	(1.96–6.53)
	14	64	45.3	23.4	18.8	12.5	16.2	**	0.29	large	2.45	*	(1.12–5.36)
	15	47	55.3	12.8	21.3	10.6	24.9	**	0.42	large	3.31	*	(1.27–8.61)
	8–15	771	45.7	26.5	18.8	9.1	227.6	**	0.31	large	3.34	*	(2.58–4.32)

Q1 to Q4 = Quartile 1 to 4; χ2 = Chi-Square Value; V = Cramer's V; P = Significance;

*P<0.05;

**P<0.01; OR = Odds ratio; 95% CI = 95% Confidence Interval.

### Part 1—data analysis

For each annual age-group, chi-square tests assessed differences between the observed and expected relative age distributions. Post hoc tests determined differences in frequency counts between significant quartiles, and the magnitude of the effect size was measured using Cramer’s *V*. For *df* = 3 which is the case for all comparisons of relative age quartiles, 0.06 < *V* ≤ 0.17 indicates a small effect, 0.17 < *V* < 0.29 a medium effect, and, *V* ≥ 0.29 a large effect. Odds Ratios (OR) and matching 95% Confidence Intervals (CI) were also calculated between Q1 and Q4 to provide an indicator of effect size.

### Part 2—procedures

For part two, in quantifying the relationships between relative and chronological age and sprint performance all data on the sample of athletes was utilised, specifically their exact decimal age in years and days old at the time for when competing at a sprint event and the electronically measured sprint time.

### Part 2—data analyses

In the first step, a linear regression using sprint time (race performance in seconds and milliseconds) as the dependent variable and decimal age as the independent variable for each annual age group was conducted with Pearson’s correlation coefficient (*r*), adjusted coefficients of determination (*R* ^*2*^), standard errors of the estimate (*SEE*) and analysis of variance calculated. Mahalanobis distances checked for the presence of outliers in the dataset using standard z-distribution cut-offs; no outliers were identified. Residuals were examined for normality, linearity, independence and homoscedasticity. All statistical assumptions for linear regression were met. The magnitude of the correlation coefficient of the regressions was initially qualitatively assessed, according to Hopkins [27] as follows: trivial *r* < 0.1, small 0.1 < *r* < 0.3, moderate 0.3 < *r* < 0.5, large 0.5 < *r* < 0.7, very large 0.7 < *r* < 0.9, nearly perfect *r* > 0.9 and perfect *r* = 1.

### Expected performance differences within and across age-groups

Mean expected performance differences per day, per month, per quartile, and per year were calculated using respective regression equations in each annual-age-group,. For example, regressions (see results) indicated that the relatively oldest consistently had the fastest expected sprint time in any given age group, while the relatively youngest were generally expected to have the slowest sprint time. A sprinter born on January 1^st^ (e.g., 8.99 in the Under 9’s) was therefore theoretically expected to be the fastest and a sprinter born on 31^st^ December is expected to be the slowest. The difference between expected sprint times of a sprinter born on January 1^st^ and on 31^st^ December provided the expected performance difference per year (i.e., 1036.8 ms—Under 9’s). As the regressions were linear, we then used the mean difference values of one year to calculate expected differences per day (by dividing by 365), quartile (by dividing by 4) and month (by dividing by 12). From these values all percentage differences were calculated.

### Corrective adjustments

To test whether corrective adjustments could remove RAEs from across the sample and at various performance levels (i.e. Top 50%, 25% and 10%), the linear regressions were used, as described in *Part 2—Data analyses*. Raw (actual) sprint times were then adjusted to account for the influence of relative age with January 1^st^ of each age group acting as the reference. For example, in the Under 9’s a person born on January 2^nd^ had their sprint time reduced by 2.84 ms; January 3^rd^ = 2.84 x 2 etc.; until December 31^st^ = 2.84 x 365 = 1036.8 ms. This process thus generated a correctively adjusted sprint time for all participants in their respective annual age-group (i.e., data from Table 2 and 3). With corrective adjustments were applied, distributions of who made the Top 50%, 25% and 10% of sprint times within each annual age group were re-examined using similar steps as that reported in Part 1—Data analysis.

**Table 2:** Linear regression equations and statistics for each annual-age group.

Age	Equation	*r*	*R* ^*2*^	SEE	*P*	Magnitude
8	y = -1.036x+19.649	0.379	0.144	0.694	**	moderate
9	y = -0.852x+18.605	0.335	0.112	0.696	**	moderate
10	y = -0.668x+17.101	0.303	0.092	0.602	**	moderate
11	y = -0.608x+16.770	0.275	0.076	0.604	**	moderate
12	y = -0.556x+16.446	0.255	0.065	0.601	**	small
13	y = -0.552x+16.525	0.265	0.070	0.576	**	small
14	y = -0.555x+16.625	0.295	0.087	0.524	**	small
15	y = -0.424x+14.741	0.283	0.080	0.437	**	small

*r* = correlation coefficient; *R* ^2^ = adjusted coefficient of determination; SEE = standard error of estimate; *P* = Significance;

*P<0.05;

**P<0.01.

**Table 3:** Mean expected performance differences (i.e., milliseconds & percentage figures) according to day, month, and quartile for each annual-age category.

Age	Δ day (ms)	Δ month (ms)	Δ Q (ms)	Δ year (ms)	Δ day (%)	Δ month (%)	Δ Q (%)	Δ year (%)
8	2.84	86.40	259.20	1036.80	0.028	0.837	2.512	10.049
9	2.33	70.97	212.90	851.61	0.023	0.703	2.110	8.441
10	1.83	55.63	166.89	667.55	0.019	0.570	1.710	6.841
11	1.67	50.69	152.06	608.26	0.018	0.535	1.606	6.422
12	1.52	46.37	139.12	556.48	0.017	0.503	1.510	6.041
13	1.51	46.00	138.00	552.00	0.017	0.523	1.569	6.275
14	1.52	46.23	138.69	554.77	0.018	0.557	1.670	6.681
15	1.16	35.32	105.97	423.86	0.015	0.444	1.331	5.326

Δ = mean expected performance difference in age group; ms = millisecond; Q = quartile.

## Results

### Part 1

Table 1 shows the quartile distributions, chi-square, effect size estimation, including ORs (and 95% CIs) for all male participants in official local, regional, or national trial events in the 2013 competitive season sub-divided according to age group and our assigned performance levels. Results identify small but significant RAEs for all age groups (except the 13 year age group which was close to significance) when all athletes were included, and when compared against the Swiss national birth distributions for each respective year. ORs progressively decreased from 2.11 at age 8 to 1.21 at age 13, before increasing again at age 14 to 1.46 and to 1.55 at age 15 respectively. However, when looking at higher performance levels, such as the fastest Top 50% of athletes, RAEs increased markedly; showing higher effect sizes (ranging from 0.15 to 0.28) and higher ORs (ranging from 1.80 to 4.55) across all age categories. This trend continued for the fastest Top 25% of athletes, revealing medium to large effect sizes and ORs ranging from 2.86 to 5.25. Finally, the highest RAEs appeared in the fastest Top 10% of athletes, with large effect sizes in all age groups with ORs significant ranging from 3.57 to 7.01 (see Table 1).

### Part 2

**Fig 1:**
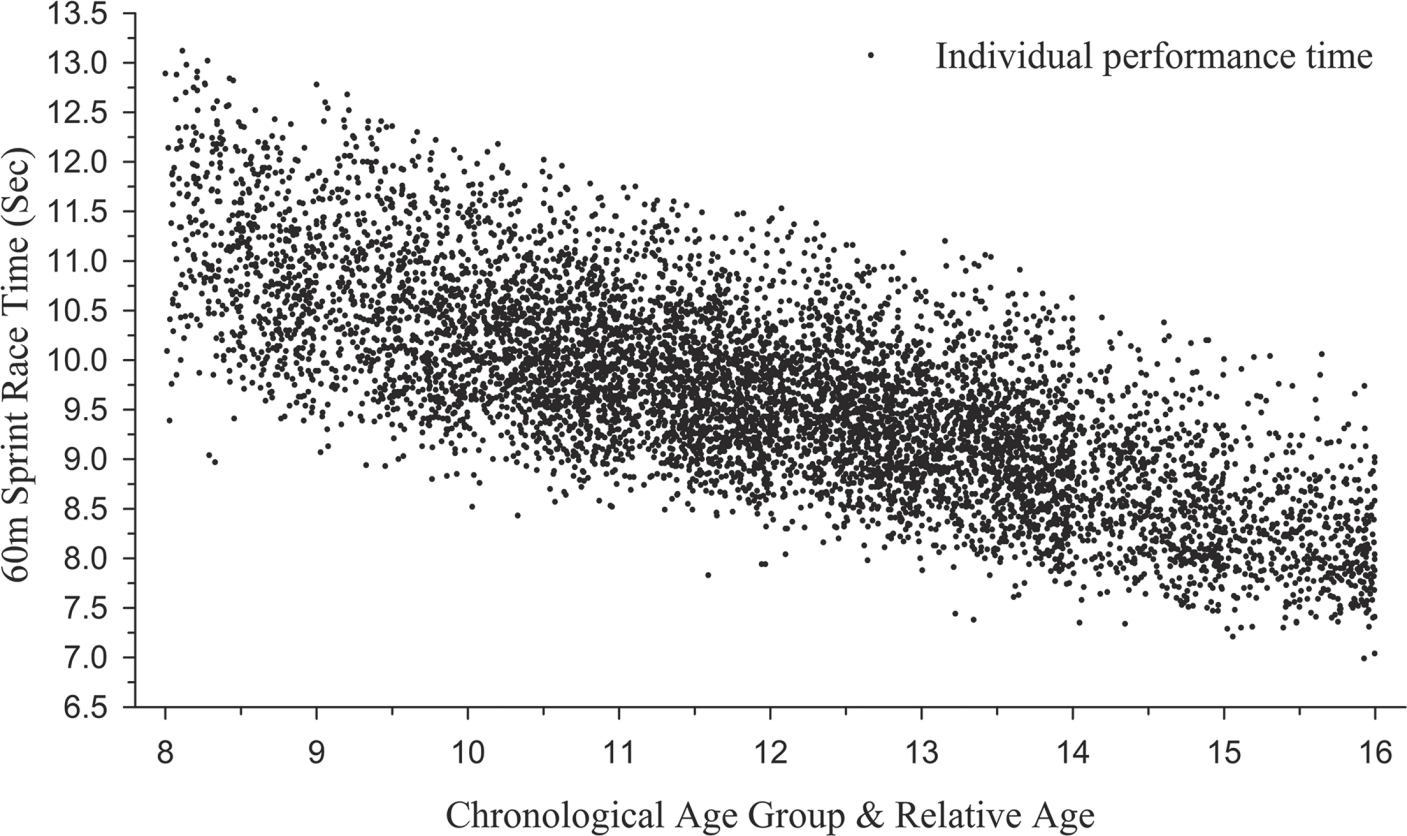
Raw 60m sprint race time performance according to chronological and relative age.

Fig 1 illustrates the relationship between 60m sprint performance according to relative and chronological age for every participant in the sample. The linear regressions within each annual age group suggest that significant proportions of the total variation of sprint performance are predicted by relative age (Table 2). Equations represent the estimated sprint time of a child or youth athlete using their exact decimal age (i.e., in years and days old) as the independent variable. Moderate to small correlations between decimal age and observed sprint times (i.e., *r* = 0.379–0.283) are shown [27]. *R* ^*2*^ indicates that approximately 14% of the variation in sprint times at the 8 year old age group was predicted by decimal age, which then decreased in subsequent age groups accounting for 8% of the variation in the 15 year olds.

### Performance differences within and across age-groups

Mean performance differences per day, month, quartile, and year are shown in Table 3. The estimated maximal performance difference in an 8 year old (i.e., Under 9’s age group) was 104 ms or 10.1% per year, 2.5% per Quartile, 0.84% per month, and 0.03% per day. Performance differences within one year decreased consistently across the age groups, until at the Under 13’s the difference between times was 55 ms or 6.3% per year, 1.6% per Quartile, 1.7% per month, or 0.02% per day. The Under 14’s differences were comparable to the Under 13’s but again decreased at the Under 15’s age group (Table 3).

### Corrective adjustments

When corrective adjustments were applied to raw sprint times and the distributions of performance levels re-examined, more equal relative age distributions (i.e., Q1—Q4) in each age-category and according to performance level were predominantly identified. For example, the corrected Top 10% in the 10 year age group showed no RAE (*p* > 0.05) and an OR of 1.05 (CI = 0.63–1.77), whereas in the original non-corrected data at the same age and performance level, a large RAE was evident (OR = 6.27, CI = 2.97–13.22). Table 4 summarises the distribution of athletes in the Top 50%, 25% and 10% following corrective adjustments. The table shows that for almost every age group in the corrected Top 50% sample, and for all age groups in both the corrected Top 25% and corrected Top 10% no significant RAEs remained (*p* > 0.05). Only in isolated cases, did small RAEs remain evident for specific age categories and performance levels (e.g., Under 9 & 10’s in the corrected Top 50%) and when all age groups were included together (i.e., 8–15 year olds).

**Table 4:** RAE prevalence within annual age-group categories after corrective adjustment.

Performance Level	Age Group	*n*	Q1%	Q2%	Q3%	Q4%	*χ* ^*2*^	*P*	V	Effect	OR	*P*	95% CI
top 50% corrected	8	288	30.6	26.7	24.7	18.1	9.0	*	0.10	small	1.66	*	(1.18–2.35)
	9	412	29.9	25.7	25.5	18.9	9.3	*	0.09	small	1.55	*	(1.17–2.06)
	10	633	27.2	28.1	25.9	18.8	11.2		0.08	no	1.42	*	(1.12–1.8)
	11	685	27.4	25.8	26.7	20.0	7.2		0.06	no	1.35	*	(1.08–1.69)
	12	678	28.5	25.4	24.5	21.7	6.5		0.06	no	1.29	*	(1.04–1.60)
	13	619	27.0	25.8	22.3	24.9	5.1		0.05	no	1.07		(0.86–1.33)
	14	324	30.2	24.4	22.5	22.8	6.1		0.08	no	1.30		(0.96–1.76)
	15	239	30.1	23.0	21.8	25.1	5.2		0.09	no	1.18		(0.84–1.66)
	8–15	3878	28.3	26.0	24.5	21.2	40.2	**	0.06	small	1.32	*	(1.20–1.45)
top 25% corrected	8	144	29.2	28.5	25.0	17.4	4.6		0.10	no	1.65	*	(1.01–2.71)
	9	206	28.2	25.7	27.7	18.4	4.0		0.08	no	1.50		(0.99–2.26)
	10	316	24.4	29.7	27.2	18.7	6.6		0.08	no	1.28		(0.91–1.80)
	11	342	21.6	28.9	28.7	20.8	5.6		0.07	no	1.03		(0.74–1.42)
	12	339	27.4	25.4	24.8	22.4	1.7		0.04	no	1.20		(0.89–1.63)
	13	309	29.4	25.9	20.7	23.9	6.5		0.08	no	1.21		(0.89–1.65)
	14	162	29.0	24.7	21.6	24.7	2.6		0.07	no	1.16		(0.76–1.76)
	15	119	30.3	19.3	21.8	28.6	5.0		0.12	no	1.04		(0.65–1.67)
	8–15	1937	26.6	26.8	25.2	21.3	11.9	**	0.05	no	1.23	*	(1.07–1.40)
top 10% corrected	8	57	22.8	28.1	24.6	24.6	0.3		0.04	no	0.91		(0.43–1.94)
	9	82	22.0	24.4	26.8	26.8	0.5		0.05	no	0.80		(0.43–1.50)
	10	126	23.8	28.6	25.4	22.2	0.8		0.05	no	1.05		(0.63–1.77)
	11	137	24.1	25.5	27.7	22.6	0.3		0.03	no	1.05		(0.64–1.71)
	12	135	26.7	24.4	26.7	22.2	0.5		0.03	no	1.18		(0.73–1.92)
	13	123	26.8	24.4	25.2	23.6	0.3		0.03	no	1.12		(0.68–1.85)
	14	64	23.4	25.0	26.6	25.0	0.1		0.02	no	0.92		(0.46–1.87)
	15	47	23.4	23.4	25.5	27.7	0.3		0.05	no	0.83		(0.37–1.86)
	8–15	771	24.5	25.6	26.2	23.7	1.0		0.02	no	1.02		(0.83–1.25)

Q1 to Q4 = Quartile 1 to 4; χ2 = Chi-Square Value; V = Cramer's V; *P* = Significance;

*P<0.05;

**P<0.01; OR = Odds ratio; 95% CI = 95% Confidence Interval.

## Discussion

Given the need to isolate and understand the mechanisms driving RAEs, and identify context appropriate solutions, researchers have highlighted the importance of broadening the scope of RAE investigations, notably to include physical demanding individual sport contexts. This study fulfilled these requirements, firstly assessing RAE prevalence across childhood and youth 60m sprinting, and by examining whether RAEs increased at higher performance levels. Secondly, it quantified the relationships between relative and chronological age with sprint performance, and tested whether a corrective adjustment procedure, which corrected for the influence of relative age at each chronological age group, identified a potential solution for RAE removal in sprinting.

### Part 1

Aligned with recent studies in individual sport contexts, small to large RAEs across childhood and youth sprinting were detected [12, 13]. Further, results determined that variations in RAE effect size were associated with annual age group and performance level characteristics. When comparing between annual age-groups, RAE effect sizes progressively decreased from ages 8–13, followed by minor increases at 14–15. These findings align well to the maturation-selection hypothesis [1], and when compared to biological growth curves showing a progressive decline in anthropometrics (e.g., height gain) by proportion, prior to a final [puberty] growth spurt, coinciding with the 13–15 age range in males [28]. Data in Fig 1 and Tables 2 and 3 also evidence the additional benefit of time for growth—reflected by higher decimal age—in the earlier years (e.g., Under 10’s) which then progressively reduce by proportion into the later years (i.e., Under 16’s).

Irrespective of annual age group, the benefit of being relatively older was clearly shown when examining the constituents of the Top 50%—Top 10% sprint performers. Similar to findings in team sport contexts [e.g.,8] RAEs and their effect sizes here increased linearly according to the performance level criteria (see Table 1), even though no formalised selection process was apparent to regulate access to higher performance levels. For instance, being relatively older substantially increased the likelihood of making the highest levels of performance in a given age group (i.e., Top 10%; e.g., Under 15’s—Q1 v Q4, OR = 3.31, CI = 1.27–8.61; across Under 8–15’s—Q1 v Q4, OR = 3.34, CI = 2.58–4.32). Thus, advanced growth remain as important necessities in attainment of higher performance levels in sprint performance; and magnified RAEs could not be attributed to social processes such selection bias *per se*.

Social processes may still exert their influence however. For instance, when all participants who voluntarily entered a 60m sprint event across Switzerland were examined, significant but small RAEs were evident across annual age-groups, suggesting that a self-selection or matching process may have occurred. The relatively older were more likely to initiate early age group sprint participation, which could possibly be based on a combination of early sporting experiences, (dis)encouraging interactions and (non)reinforcement with others (e.g., parents & peers), as well as the alignment between perceived physical capability in sprinting relative to others. Social processes may also better explain, compared to the maturation-selection hypothesis, why small RAEs remained after corrective adjustments in isolated age-groups (e.g., Under 9’s) as shown in part two of the study. For example, the greater total number of Q1’s v Q4 participants at the Under 9’s meant that it was impossible to have equal distributions even after corrective adjustments were applied to the Top 50% of sprint times, as Q1’s and Q4’s represented 33.4% and 15.6% of participants respectively (i.e., > 50% difference in numbers). In others words corrective adjustment was never going to, and neither did in intend, to totally correct for participation based RAEs.

### Part 2

Linear regressions identified that the predictive influence of decimal age on sprint speed performance were moderate to small, and that performance differences per year decreased progressively from 10.1% to 5.3% approximately between 8–15 years of age. However, unique and novel here was that mean expected performance differences could also provide corrective adjustments figures. When appropriately applied to each individuals athlete’s sprint time with January 1^st^ as the reference (i.e., relatively oldest), a re-analysis of RAE distributions according to performance level identified that corrective adjustments were capable of generally removing RAEs from Swiss 60m sprinting. RAEs in each age group of the corrected Top 25% and Top 10% of athletes became completely absent (i.e., *p* >. 05), with more even distributions across Q1-Q4 demonstrated, bar the few explained exceptions. To illustrate, in the thirteen year old age group a substantial difference existed between the fastest Top 10%, and the ‘relative age corrected’ distribution. In the fastest 10% of sprint times at that age, over 45% were from Quartile 1 reflecting a 20% overrepresentation (i.e., 45–20 = 25% per quartile) while only 11% were from Q4 indicating a 14% underrepresentation. However, after applying corrective adjustments, the corrected Top 10% included 27% from Q1 and 24% from Q4 showing no statistical RAE.

Corrective adjustments demonstrate the capability to more accurately compare between individuals, given their specific relative age, sprint times, and with comparison to a broader reference data set. For instance, in the data there was a 10 year old boy (boy 1) born on the 13^th^ of February, and a boy (boy 2) born the 18^th^ of November. Boy 1 had a race time of 8.92s, while boy 2 had a race time of 9.17. Boy 1 in real terms was 0.25s (i.e., 9.17s-8.92s) faster than boy 2. However, after adjusting sprint times (i.e., age difference of 0.87 years and expected performance difference of 5.21%), boy 1 actually had a corrected net sprint time of 8.85s, while boy 2 had a corrected net sprint time of 8.62s. This means that if the relative age advantage was correctly accounted for and adjusted, boy 2 actually had a better sprint time given their respective relative ages.

### Implications

Findings challenge present norms and practice in both grass-roots sport participation and in athlete development systems. First, due to their comparatively later biological development, a substantial majority of relatively younger athletes in childhood and youth ages are likely to perform comparatively poorly in age-based competition, and may fail to meet selection requirements for athlete developmental systems. Over time though and as our data suggests, the disadvantage is likely to diminish by proportion; and other factors are likely to become more influential [29]. Thus, it seems that RAEs reflect a type of developmental barrier; one which is preventable if appropriate solutions can be implemented.

Corrective adjustments may hold significant implications for current childhood and youth sport contexts in both team and individual CGS sport contexts [1,30], where the influence of relative age is presently not considered or removed, resulting in what are consistent and sometimes large RAE effect sizes. In Swiss track and field, corrective adjustments can help ensure that potential sprinters are not ignored, missed, or lost on the basis of relative age or later growth. For team sports such as soccer and codes of rugby, where players are often assessed on standard multiple anthropometric and physiological/fitness tests (e.g., sprint times, vertical jump), corrective adjustments could help better inform and improve validity in player evaluation and selection procedures.

Although in alternative forms, corrective adjustments do already exist in other sport contexts and disciplines. Handicapping in golf [31] is a corrective adjustment method for skill level; while in standardised physiological performance tests, oxygen uptake and force production are often normalised for body weight [32]. So testing and application of corrective adjustments in specific CGS junior/youth sports, or in contexts where components of physical performance are measured in CGS, are important future directions. Whether sport coaches, sport federations/governing bodies, and athlete development systems perceive value in implementing such procedures remains to be determined. From our standpoint, the main challenge relates to the obtainment of a substantial reference data-set to generate accurate regressions and subsequent corrective adjustments. If overcome and applied, corrective adjustment are likely to help remove RAEs from affecting sport participation experience across childhood and youth sport, and help make long-term athlete development more legitimate and effective.

## Conclusion

Overall findings identified small RAE effect sizes across age groups when all 60m sprinters were analysed. RAE effect sizes decreased as age-group increased, but regardless of age-group increased linearly according to performance level. Regression analyses between decimal age and sprint time identified that an almost one year relative age difference resulted in performance differences of 10.1% at age 8, 8.4% at 9, 6.8% at 10, 6.4% at 11, 6.0% at 12, 6.3% at 13, 6.7% at 14, and 5.3% at 15. Correction adjustments calculated according to day, month, quarter, and year showed that the influence of relative age—and thus normative growth and development—can be accounted for and RAEs removed from sprint performance. Corrective adjustments could also be considered and need to be evaluated for other disciplines in track and field (e.g., 100m+, long jump or throwing). Importantly, findings highlight a potential solution to help remove RAEs from CGS sports; help improve childhood and youth sport participation experience; and help improve inter-athlete evaluation assessment and selection.
